# Rapid Screening and Identification of Daidzein Metabolites in Rats Based on UHPLC-LTQ-Orbitrap Mass Spectrometry Coupled with Data-Mining Technologies

**DOI:** 10.3390/molecules23010151

**Published:** 2018-01-12

**Authors:** Wenjing Zhao, Zhanpeng Shang, Qinqing Li, Moran Huang, Wenbin He, Zhibin Wang, Jiayu Zhang

**Affiliations:** 1 School of Chinese Pharmacy, Beijing University of Chinese Medicine, Beijing 100102, China; cosyzwj@126.com (W.Z.); zpshang1206@163.com (Z.S.); 15810152609@163.com (M.H.); 2 Shanxi Key Laboratory of Chinese Medicine Encephalopathy, Shanxi University of Chinese Medicine, Jinzhong 030619, China; lqqlqq@126.com (Q.L.); hewenbinbin@aliyun.com (W.H.); 3 Beijing Research Institute of Chinese Medicine, Beijing University of Chinese Medicine, Beijing 100029, China

**Keywords:** daidzein, metabolic profiling, UHPLC-LTQ-Orbitrap mass spectrometry, data-mining technologies

## Abstract

Daidzein, the main bioactive soy isoflavone in Nature, has been found to possess many biological functions. It has been investigated in particular as a phytoestrogen owing to the similarity of its structure with that of the human hormone estrogen. Due to the lack of comprehensive studies on daidzein metabolism, further research is still required to clarify its in vivo metabolic fate and intermediate processes. In this study, an efficient strategy was established using UHPLC-LTQ-Orbitrap mass spectrometry to profile the metabolism of daidzein in rats. Meanwhile, multiple data-mining methods including high-resolution extracted ion chromatogram (HREIC), multiple mass defect filtering (MMDF), neutral loss fragment (NLF), and diagnostic product ion (DPI) were utilized to investigate daidzein metabolites from the HR-ESI-MS^1^ to ESI-MS*^n^* stage in both positive and negative ion modes. Consequently, 59 metabolites, including prototype compounds, were positively or tentatively elucidated based on reference standards, accurate mass measurements, mass fragmentation behaviors, chromatographic retention times, and corresponding calculated Clog*P* values. As a result, dehydration, hydrogenation, methylation, dimethylation, glucuronidation, glucosylation, sulfonation, ring-cleavage, and their composite reactions were ascertained to interpret its in vivo biotransformation. Overall, our results not only revealed the potential pharmacodynamics forms of daidzein, but also aid in establishing a practical strategy for rapid screening and identifying metabolites of natural compounds.

## 1. Introduction

Daidzein (4′,7-dihydroxyisoflavone), one of the most prominent soy isoflavones, is largely restricted to leguminous plants, such as *Trifolium pretense* L., *Medicago sativa* L. and *Pueraria lobata* Ohwi [1,2]. In recent years, the observed health benefits and versatile pharmacological properties of daidzein, including its anti-cancer (anti-breast cancer and anti-prostate cancer), anti-cardiovascular disease, anti-osteoporosis, anti-diabetic, anti-aging, anti-oxidant, and anti-inflammatory activities have been extensively investigated [3,4,5,6]. In addition, daidzein is also reported to exhibit various bio-activities against dermatosis and neurodegenerative diseases [7,8,9,10]. However, until now there are no comprehensive studies focused on its in vivo metabolism, which is important for revealing its pharmacologically active substances.

In the past decades, metabolism studies were normally initiated when a molecule had cleared the discovery process and entered the development phase, and they were usually achieved using liquid chromatography/ultraviolet and visible spectroscopy (LC/UV) and gas chromatography/mass spectrometry (GC/MS). Especially, the preponderance of liquid chromatography coupled with high resolution mass spectrometry (LC-HR-MS), such as LTQ-Orbitrap MS, FT-ICR-MS, etc., in the structural characterization of known and unknown metabolites due to the properties of high speed, efficiency, selectivity and detection sensitivity has been fully proved by disparate research teams [11,12,13]. Besides, it can also provide precise elemental composition from accurate mass measurement, which is tremendously helpful to identify of major-to-minor metabolites in vitro and in vivo [14]. Accordingly, drug metabolism can be fully presented with high degrees of certainty even in situations where the corresponding reference standards are lacking. Thus, it is feasible to establish a comprehensive and integrated analytical workflow-interpretation sequence to obtain useful information from complex backgrounds [15,16,17,18]. Recently, efficient data-mining methods including isotope pattern filtering (IPF) [19], diagnostic product ion (DPI) [15,20], neutral loss filtering (NLF) [21], extracted ion chromatogram (EIC) [22], mass defect filter (MDF), and multiple mass defect filters (MMDFs) [23] have been successful applied to systematically profile the in vivo drug metabolism of drugs. 

Herein, a high sensitive and specific UHPLC-LTQ-Orbitrap MS based method in both negative and positive ion modes with multiple data-mining methods was established to profile and identify the major-to-minor metabolites in Sprague-Dawley (SD) rats after oral administration of daidzein. Meanwhile, the potential metabolic pathways of daidzein were also proposed in this study. To our best knowledge, it is the first time to the in vivo metabolism of daidzein has been comprehensively investigated.

## 2. Results

### 2.1. Establishment of the Analytical Workflow-Interpretation Method

**Figure 1:**
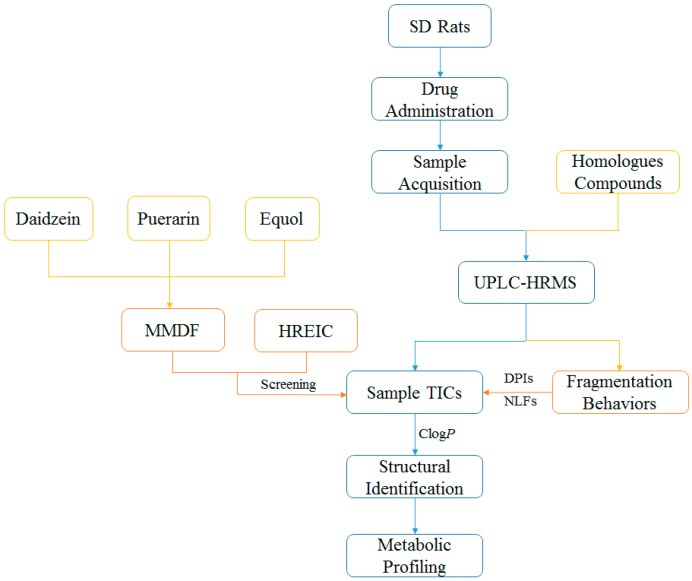
Summary diagram of the developed strategy and methodology.

A novel and integrated strategy (Figure 1) was established for profiling the in vivo daidzein metabolism based on UHPLC-LTQ-Orbitrap MS coupled with multiple post-acquisition data-mining methods. First, the reported metabolites of isoflavones were summarized via literature searches to ascertain the necessary reference standards. Secondly, an ESI-MS*^n^* dataset of samples and five selected reference standards were obtained in data-dependent scan (DDS) acquisition mode. After that, HREIC and MMDF were used to screen the daidzein metabolite candidates at the HR-MS^1^ level. Among them, HREIC was employed to ascertain the known and predicted metabolites, while MMDF was utilized to obtain HR-MS^1^ special information of the unknown and unpredicted metabolites. Then, the PIL-DE data-acquisition method was adopted to obtain the ESI-MS*^n^* datasets of those screened metabolite candidates. Afterwards, the potential structures of daidzein metabolites were expounded in accordance with reference standards, chromatographic retention times, accurate mass measurement, the proposed DPIs and NLFs (summarized from the mass fragmentation behaviors of reference standards), and the corresponding calculated Clog*P* values. Along with the established strategy, daidzein metabolites would be positively or tentatively identified and the corresponding metabolic pathways were also proposed.

### 2.2. The Establishment of MMDF Approach

In order to obtain the special HR-MS^1^ datasets of common-to-uncommon and major-to-minor metabolites accurately and comprehensively, the MMDF approach was implemented as a complement to the HREIC method. For an MMDF method, in the case of negative ion mode, first and foremost, according the results of literature studies and HREIC searches, three common metabolites (daidzein, daidzin, and equol) were selected to be MDF templates, which covered drug and conjugation filters. The second step was to confirm the mass range and mass defect range based on the substitution of various templates. As a result, each MDF window was frequently set to ±50 mDa around the mass losses of the templates over a mass range of ±50 Da around the filter template masses. Therefore, the parent drug filter templates were set as follows: (1) parent drug template (*m*/*z* 253.0494) and its conjugation templates (*m*/*z* 335.0219 for sulfate conjugation and *m*/*z* 429.0815 for glucuronide conjugation); (2) puerarin template (*m*/*z* 415.1023) and its conjugation templates (*m*/*z* 495.0591 for sulfate conjugation and *m*/*z* 591.1344 for glucuronide conjugation); (3) equol template (*m*/*z* 241.0858) and its conjugation templates (*m*/*z* 321.0427 for sulfate conjugation and *m*/*z* 417.1179 for glucuronide conjugation). Based on this efficient method, even the heterogeneous ions exist, minor metabolites can also be screened out from the complex background noise and endogenous components.

### 2.3. Mass Fragmentation Behavior Analyses of Daidzein and Its Homologues

**Figure 2:**
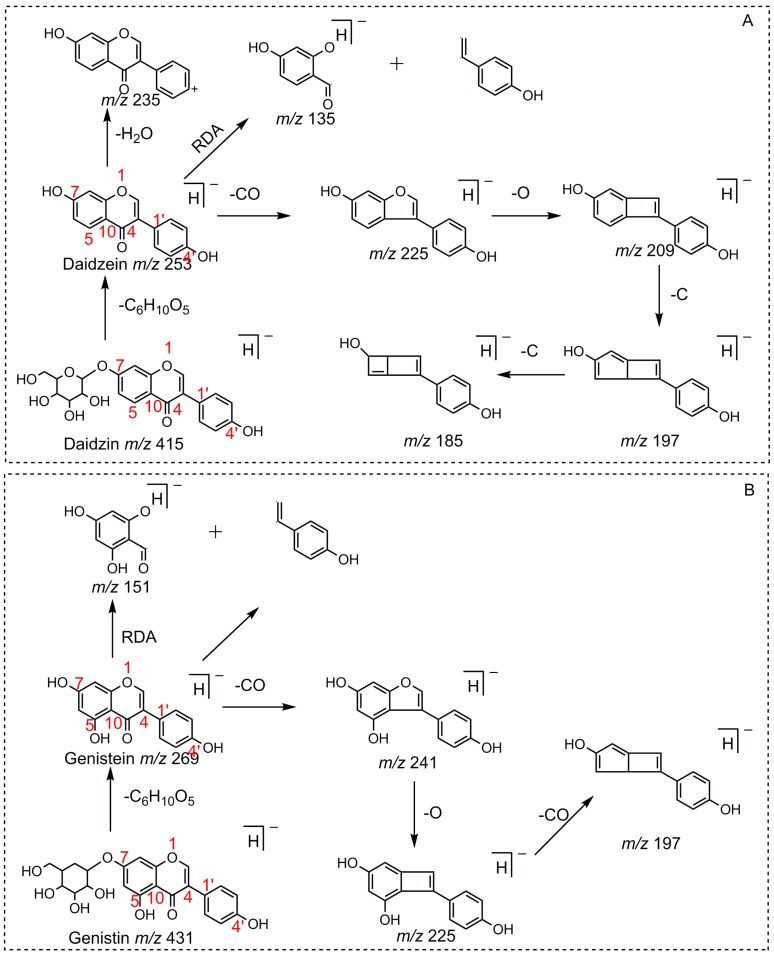
The mass fragmentation behavior of daidzein, daidzein, genistein, and genistin in negative ion mode ((**A**) daidzin and daidzein; (**B**) genistin and genistein; Red number is location for compound nomenclature).

For a better understanding of the ESI-MS*^n^* fragmentation patterns of daidzein and the other four reference standards (daidzin, genistein, genistin, and puerarin), the mixed standard solution was continuously analyzed by UHPLC-LTQ-Orbitrap MS. Taking daidzein in negative ion mode for example, it showed the [M−H]^−^ ion at *m*/*z* 253.0495 (C_15_H_9_O_4_, 0.29 ppm) in ESI-MS^1^ spectrum. Several characteristic product ions at *m*/*z* 225.0556 (C_14_H_9_O_3_, 1.01 ppm), *m*/*z* 224.0480 (C_14_H_8_O_3_, 1.26 ppm), *m*/*z* 197.0606 (C_13_H_9_O_2_, 0.92 ppm), *m*/*z* 185.0606 (C_12_H_9_O_2_, 0.99 ppm), and *m*/*z* 135.0086 (C_7_H_3_O_3_, 0.95 ppm) were respectively generated by loss of CO, CHO, CO_2_, 2CO, 2CO + C, and C_8_H_6_O. In particular, a successive neutral loss of CO (*m*/*z* 225.0556 and *m*/*z* 197.0606) could be considered as the distinctive fragmentation behavior of soy isoflavones to implement the rapid metabolite identification. The proposed mass fragmentation patterns of daidzein were illustrated in Figure 2. Daidzin, genistein, and genistin possessed similar mass fragmentation patterns to those of daidzein, which were also illustrated in Figure 2 [24]. In addition, the corresponding mass fragmentation behaviors in positive ion mode are shown in Figure S1.

**Figure 3:**
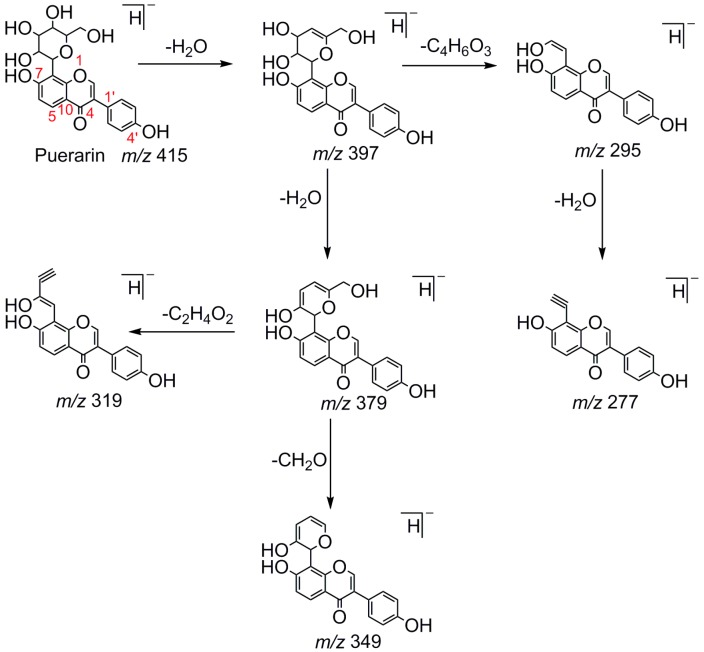
The mass fragmentation behavior of puerarin (Red number is location for compound nomenclature).

Puerarin, a common C-glycoside, possessed specific fragmentation behaviors, which were totally different from the other four reference standards. It generated the [M−H]^−^ ion at *m*/*z* 415.1059 (C_21_H_19_O_9_, 3.51 ppm) in the ESI-MS spectrum in negative ion mode. In the ESI-MS^2^ spectrum, it yielded a series of product ions which occurred on heteroside moiety by neutral loss of H_2_O, 2H_2_O, 2H_2_O + CH_2_O, 2H_2_O + 2CH_2_O, 2H_2_O + 2CH_2_O + C, 2H_2_O + 2CH_2_O + 2C, and 3H_2_O + 2CH_2_O + 2C, including *m*/*z* 397.0917 (C_21_H_17_O_8_, 0.82 ppm), *m*/*z* 379.0812 (C_21_H_15_O_7_, 1.14 ppm), *m*/*z* 349.0706 (C_20_H_13_O_6_, 0.79 ppm), *m*/*z* 319.0600 (C_19_H_11_O_5_, 1.01 ppm), *m*/*z* 307.0600 (C_18_H_11_O_5_, 0.55 ppm), *m*/*z* 295.0600 (C_17_H_11_O_5_, 2.07 ppm), and *m*/*z* 277.0495 (C_17_H_9_O_4_, 0.44 ppm) [25]. Among them, a successive neutral loss of water moiety could be employed as the characteristic of *C*-glycoside based structures. Especially, the neutral loss of 120 Da (*m*/*z* 415.1059 to *m*/*z* 295.0600) could also be utilized to differentiate *C*-glycoside metabolites. The proposed mass fragmentation patterns of puerarin in negative ion mode were illustrated in Figure 3 and the corresponding spectra in positive ion mode are shown in Figure S1C.

Compounds with similar substructures will exhibit similar fragmentation behaviors in the ESI-MS*^n^* spectra, and thus produce certain common DPIs and regular NLFs. In various complex matrices, ascertaining the DPIs could facilitate the rapid and comprehensive identification of drug metabolites. For example, the DPI at *m*/*z* 135, which was diagnostically owing to Retro-Diels-Alder (RDA) rearrangement from the 1,4-position in the C-ring of daidzein, would provide information about whether bioreactions occurred on the A-ring or not. Thus, in the ESI-MS^2^ spectra of daidzein metabolites, the occurrence of DPIs at *m*/*z* 135 or *m*/*z* 135 + X (X = mass weight of substituent groups, such as 14, 16, 80, 162, 176, etc.) gave the information similar to that described above. For example, in the MS^2^ spectrum of genistein, DPI at *m*/*z* 151, which was 16 Da more than the DPI at *m*/*z* 135.0086 yielded by daidzein, further validated the above deduction. In addition, NLF could provide much more information for the structural elucidation. For example, the successive NLFs of 28 Da (CO) in the ESI-MS*^n^* spectra of daidzein and 120 Da (C_4_H_8_O_4_) in ESI-MS*^n^* spectra of puerarin also provided tremendous help to identify these metabolites.

### 2.4. Identification of Daidzein Metabolites in Rats

**Figure 4:**
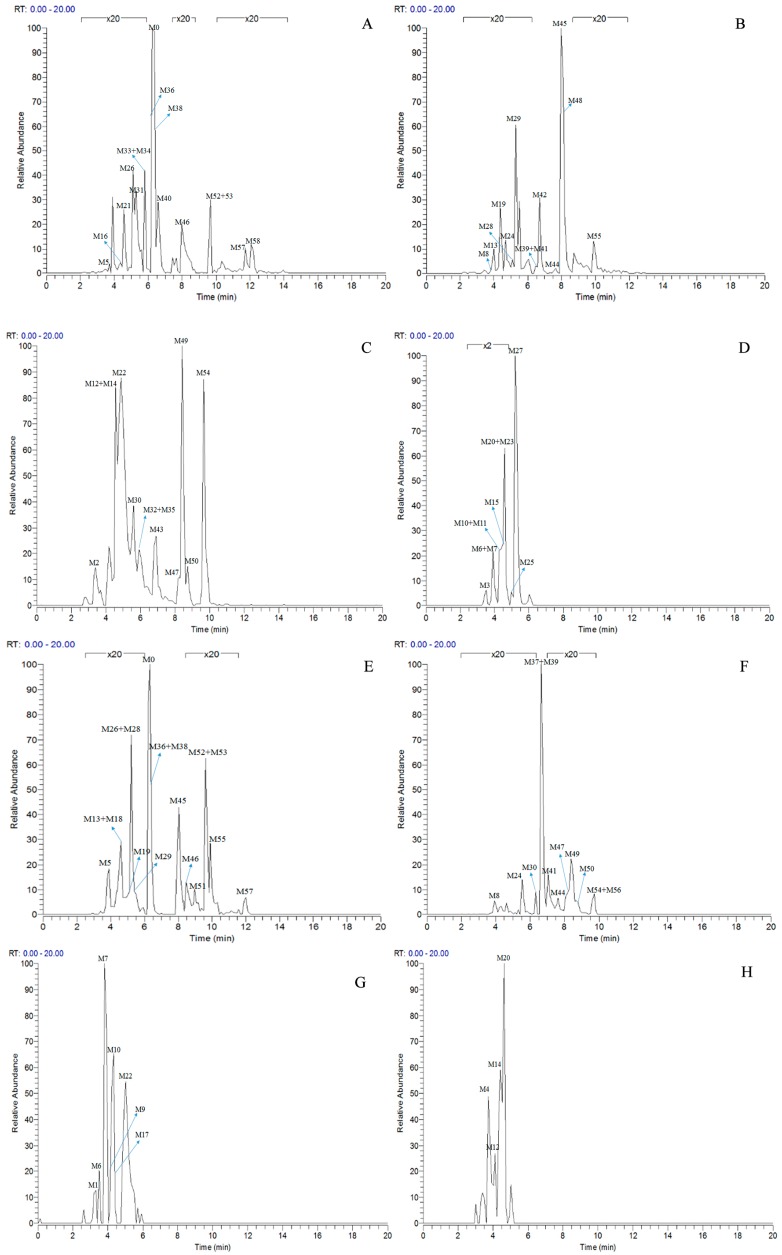
High resolution extracted ion chromatograms for the multiple daidzein metabolites in ±5 ppm error ((**A**–**D**) for negative ion mode, (**E**–**H**) for positive ion mode): (**A**) *m*/*z* 225.0546, 239.0702, 241.0858, 253.0495, 255.0652, 257.0808; (**B**) *m*/*z* 267.0652, 269.0444, 271.0600, 283.0600, 285.0393, 285.0757; (**C**) *m*/*z* 287.0913, 299.0550, 333.0063, 335.0219, 337.0376, 593.1506, 595.1657; (**D**) *m*/*z* 415.1023, 417.1180, 431.0983, 447.0931; (**E**) *m*/*z* 227.07019, 241.0854, 243.1015, 255.0657, 257.0808, 259.0959, 269.0808, 271.0600; (**F**) *m*/*z* 273.0757, 275.0908, 285.0756, 287.0544, 289.1071, 299.0920, 301.0701; (**G**) *m*/*z* 335.0219, 417.1179, 431.0972; (**H**) *m*/*z* 433.1129, 579.1708, 595.1657.

According to total ion chromatograms (TICs) provided by Xcalibur 2.1, the FS mass spectra of rat urine and plasma samples after oral administration of daidzein were respectively compared with control ones for the identification of metabolites. Processing the data acquired by the UHPLC-LTQ-Orbitrap instrument led to the discovery of 59 metabolites in both negative and positive ion modes. Among them, 40 metabolites were found in positive ion mode, while 50 metabolites were found in negative ion mode. All the related mass data was summarized in Table 1, and HREICs of daidzein metabolites are shown in Figure 4.

#### 2.4.1. Identification of Isoflavone Metabolites

Metabolites M0, M5, M21, and M57, which possessed the same [M−H]^−^ ions at *m*/*z* 253.0495 (C_15_H_9_O_4_, error ≤ ±2.00 ppm), were respectively eluted at 3.85, 4.87, 6.30 and 11.76 min. By comparing the retention time and fragmentation behaviors with a daidzein reference standard, M0 was unequivocally interpreted to be unchanged daidzein parent compound. M5, M21 and M57 were consistent with M0 in accurate mass weight and product ions (*m*/*z* 225, *m*/*z* 224, *m*/*z* 197, *m*/*z* 185 and *m*/*z* 135), indicating that all of them could be identified as the positional isomers of daidzein generated by in vivo biotransformation.

Metabolite M55 eluted at 9.93 min showed its [M−H]^−^ ion at *m*/*z* 267.0660 (C_16_H_11_O_4_, 0.89 ppm), and then yielded DPI at *m*/*z* 252 via the neutral loss of 15 Da (CH_3_ moiety), which indicated that M55 was methylated product of daidzein. In addition, another DPI at *m*/*z* 134 (yielded by RDA rearrangement occurred on positions 1 and 3) further indicated that the methylation occurred on A-ring. Therefore, M55 was identified as 7-*O*-methydaidzein [26].

Metabolites M1, M9, M17, and M22, respectively eluted at 3.36, 4.13, 4.56, and 4.87 min, gave rise to the same [M + H]^+^ ions at *m*/*z* 335.0219 (C_15_H_11_O_7_S, error ≤ ±1.50 ppm). Their ESI-MS*^n^* spectra all showed the DPI at *m*/*z* 255 ([M + H-SO_3_]^−^) corresponding to a NLF of 80 Da from the parent ion at *m*/*z* 335, which could be aided us to preliminarily deduce them as sulfonation products of daidzein. A series of product ions at *m*/*z* 225, *m*/*z* 224, and *m*/*z* 197 provided adequate evidences for our deduction. Therefore, they were tentatively inferred to be daidzein-*O*-sulfate [27].

Metabolite M6 eluted at 3.85 min gave rise to the [M−H]^−^ ion at *m*/*z* 415.1031 (C_21_H_19_O_9_, 0.76 ppm), which was 162 Da more than that of daidzein. In its ESI-MS^2^ spectrum, M6 exhibited a NLF of 162 Da to yield [M-H-Glu]^−^ ion at *m*/*z* 253, which attributed to the presence of a glucosyl group in its structure. By comparing the retention time and mass fragmentation behavior with reference standard, M6 was unambiguously assigned as daidzin [28]. Metabolite M10 generated the [M−H]^−^ ion at *m*/*z* 415.1023 (C_21_H_19_O_9_, 0.00 ppm). Its ESI-MS*^n^* spectra were the same as those of daidzin, which indicated it might be daidzin isomer. Thus, M10 was tentatively identified to be daidzein-4′-*O*-glucoside due to the existing hydroxyl group in the C-ring. Meanwhile, in the ESI-MS*^n^* spectra of M4, NLFs of 162 Da (from *m*/*z* 579 to *m*/*z* 415) and 324 Da (from *m*/*z* 415 to *m*/*z* 253) indicated it might be a glucosylation product of daidzin. The observed base peak ion at *m*/*z* 253 further indicated that glucosyl group might be introduced to the heteroside moiety. Thus, owing to the existence of various active sites in the glucosyl moiety, the actual reaction site could not be determined and M4 was tentatively identified as daidzin-*O*-glucoside.

Metabolites M7 and M11 showed retention times of 3.85 and 4.28 min, respectively. They exhibited the same molecular ion at *m*/*z* 429.0815 (C_21_H_17_O_10_, error ≤ ±1.50 ppm), which were 176 Da higher than that of daidzein. In their ESI-MS^2^ spectra, the DPI at *m*/*z* 253 resulted from a neutral loss of a glucuronide moiety. According to the structure of daidzein, there are two possible conjugation sites which could form positional isomers with identical mass weights. Therefore, M7 and M11 were respectively characterized as daidzein-7-*O*-glucuronide (Clog*P*, −0.10) and daidzein-4′-*O*-glucuronide (Clog*P*, 0.08) [1].

Metabolites M13, M19, M28, M29, and M45 were eluted at 4.37, 4.61, 5.27, 5.51, and 8.05 min, respectively. All of them gave rise to [M−H]^−^ ions at *m*/*z* 269.0444 (C_15_H_9_O_5_, error ≤ ±1.00 ppm), which were 16 Da more than that of M0. In their ESI-MS^2^ spectra, DPI at *m*/*z* 151 that were also 16 Da more than DPI at *m*/*z* 135 yielded by daidzein, indicated that a hydroxyl group was introduced to the A-ring. Among those metabolites, M45 showed a retention time precisely matching that of a reference standard of genistein, thus, M45 was positively identified as genistein, while M13, M19, M28, and M29 were tentatively proposed as positional isomers of genistein [29,30].

Metabolites M8, M24, and M41 were extracted in the HREIC at *m*/*z* 283.0608 (C_16_H_11_O_5_, error ≤ ±1.00 ppm) in negative ion mode with retention times at 3.97, 4.94, and 6.62 min. They were 14 Da more than that of genistein, and the DPI at *m*/*z* 268 [M-H-CH_3_]^−^ indicated that they might be methylated products of genistein. The DPI at *m*/*z* 165 (yielded by RDA rearrangement occurred on positions 1 and 3) of M8 and M41 further indicated that the methyl group was introduced to the A-ring. On the contrary, DPI at *m*/*z* 151 (generated by RDA rearrangement occurred on positions 1 and 3) of M24 indicated that the methylation occurred on its C-ring. Therefore, M8, M24, and M41 were tentatively identified as 5-*O*-methylgenistein (Clog*P*, 2.09), 4′-*O*-methylgenistein (Clog*P*, 2.98), and 7-*O*-methylgenistein (Clog*P*, 2.99) based on their chromatography retention times, mass fragmentation behaviors, and calculated Clog*P* values.

M56 showed a [M + H]^+^ ion at *m*/*z* 299.0904 (C_17_H_15_O_5_, −0.92 ppm), which was 14 Da more than that of methylgenistein. The DPIs at *m*/*z* 284 and *m*/*z* 269 by successive neutral loss of CH_3_ indicated that M56 might be a methylated product of M42. In addition, the DPI at *m*/*z* 166 owing to RDA rearrangement that occurred on positions 1 and 3 further indicated that those two methyl groups were all introduced into the A-ring, hence, M56 was tentatively identified as 5, 7-dimethygenistein.

Metabolites M30, M47, M49, and M50, whose retention times were 5.60, 8.24, 8.44 and 8.74 min, generated the same [M−H]^−^ ions at *m*/*z* 299.0550 (C_16_H_11_O_6_, error ≤ ±1.00 ppm). [M-H-CH_3_]^−^ at *m*/*z* 284 was displayed in their ESI-MS^2^ spectra without exception, indicating occurrence of methylation. In their ESI-MS^3^ spectra, the base peak ion at *m*/*z* 284 gave a prominent [M-H-CH_3_-CO]^−^ ion at *m*/*z* 256 and a [M-H-CH_3_-CO-CO]^−^ ion at *m*/*z* 228, which was in accordance with the fragmentation behavior of M45. Hence, they were tentatively identified as methylation and hydroxylation products of genistein.

Metabolites M20 and M25 eluted at 4.61 and 5.01 min and gave rise to respective [M−H]^−^ ions at *m*/*z* 431.0983 (C_21_H_19_O_10_, 1.06 ppm,) and *m*/*z* 431.0983 (C_21_H_19_O_10_, 1.03 ppm). The DPI at *m*/*z* 269 by loss of glycosyl moiety in their ESI-MS^2^ spectra, suggesting they might be glucosylation products of genistein. M20 possessed the same retention time and mass fragmentation patterns as a genistin reference standard. Therefore, M20 was unambiguously identified as genistin, whereas M25 was tentatively assigned as a genistin isomer.

Metabolites M12 and M14, eluted at 4.36 and 4.39 min, respectively, showed [M+H]^+^ ions at *m*/*z* 595.1644 (C_27_H_31_O_15_, error ≤ ±3.00 ppm). They were 162 Da more than that of genistin in positive ion mode, which were formed by addition of a glucose to genistin. In their ESI-MS^2^ spectra, DPIs at *m*/*z* 433 and *m*/*z* 271 by successive neutral loss of glucosyl moieties were observed. Thus, M12 and M14 were respectively identified as 4′-*O*-glucosygenistin (Clog*P*, −2.27) and 5-*O*-glucosygenistin (Clog*P*, −1.70).

#### 2.4.2. Identification of Puerarin Species Metabolites

Metabolite M3 eluted at 3.46 min possessed a [M−H]^−^ ion at *m*/*z* 415.1032 (C_21_H_19_O_9_, 0.88 ppm). By comparing the chromatographic retention time and MS/MS spectra, M3 was positively identified as puerarin. M39 gave rise to a [M−H]^−^ ion at *m*/*z* 285.0403 (C_15_H_9_O_6_, 0.98 ppm) with a retention time of 6.52 min. It was 16 Da more than that of genistein. Therefore it could be tentatively identified as hydroxygenistein. There were abundant product ions at *m*/*z* 257 and *m*/*z* 229 in the ESI-MS^2^/MS^3^ spectra produced by successive neutral losses of CO. Meanwhile, the DPI at *m*/*z* 151 (yielded by RDA rearrangement occurred on positions 1 and 3) further confirmed that hydroxylation occurred on B-ring. Therefore, M39 was tentatively identified as hydroxygenistein. M15 and M23 were 162 Da more than that of M39, indicating they might be glucosylation products of M39. The DPI at *m*/*z* 285 ([M-H-Glu]^−^) was yielded by neutral loss of glycosyl moiety, which indicated the presence of glucosyl group. Therefore, they were tentatively identified as hydroxygenistein-*O*-glucosides.

#### 2.4.3. Identification of Hydrogenation Isoflavone Species Metabolites

Metabolites M16, M38, and M58 with respective retention times of 4.56, 6.40 and 12.11 min, exhibited the same [M−H]^−^ ions at *m*/*z* 255.0652 (C_15_H_11_O_4_, error ≤ ±1.50 ppm), which were 2 Da more than that of daidzein. It suggested that these three metabolites could be hydrogenation products of daidzein. The DPIs at *m*/*z* 149 (generated by RDA rearrangement occurring on positions 2 and 3) and *m*/*z* 135 (yielded by RDA rearrangement occurring on positions 1 and 3) provided substantial evidence that dehydrogenation had happened to the double bond of C-ring, and thus, they were tentatively identified as hydrogenation products of daidzein.

Metabolites M31, M34, M40, and M53 possessed the same [M−H]^−^ ions at *m*/*z* 257.0808 (C_15_H_13_O_4_, error < ±1.50 ppm) with retention times at 5.70, 5.90, 6.62 and 9.65 min. They were 4 Da more than that of daidzein, which indicated that they might be dihydrogenation products of daidzein. In their ESI-MS^2^ spectra, DPIs at *m*/*z* 137 ([M-H-C_8_H_8_O]^−^, generated by RDA rearrangement occurred on positions 1 and 3) and *m*/*z* 121 ([M-H-C_8_H_8_O_2_]^−^, yielded by RDA rearrangement occurred on positions 2 and 4) of M31, M34, and M40 demonstrated that the dihydrogenation occurred on the C-ring (shown in Figure S2A,B) and thus, they were tentatively identified as dihydrogenation products of daidzein. M53 generated the same [M−H]^−^ ion with M31, M34, and M40, but different fragmentation behaviors indicated the disparate structures they represented. In its ESI-MS^2^ spectrum, DPIs at *m*/*z* 163 ([M-H-C_6_H_6_O]^−^, generated by RDA rearrangement occurred on positions 3 and 1’) and *m*/*z* 109 ([M-H-C_9_H_8_O_2_]^−^, yielded by RDA rearrangement occurred on positions 4 and 10) indicated it should be *O*-demethylangolensin (shown in Figure S2C,D).

M37 eluted at 6.39 min gave rise to a [M + H]^+^ ion at *m*/*z* 275.0908 (C_15_H_15_O_5_, −0.55 ppm). The observed DPI of *m*/*z* 151 was generated by RDA rearrangement which occurred on positions 1 and 3 in the C-ring. Therefore, M37 was tentatively identified as 3-(3,4-dihydroxyphenyl) chromane-4,7-diol.

M2, M32, and M35 were respectively eluted at 3.36, 5.70 and 5.90 min. All of them gave rise to the same [M−H]^−^ ions at *m*/*z* 337.0376 (C_15_H_13_O_7_S, error ≤ ±2.00 ppm). The DPI at *m*/*z* 257 formed by neutral loss of 80 Da indicated the presence of a sulfone group. In their ESI-MS^3^ spectra, DPIs at *m*/*z* 137 and *m*/*z* 121 were identical with those of M31, M34, and M40. Therefore, M2, M32, and M35 were respectively identified as 3-(4-hydroxyphenyl) chromane-4-ol-7-*O*-sulfate (Clog*P*, −0.20), 3-(4-*O*-sulfate phenyl) chromane-4,7-diol (Clog*P*, −0.19), and 3-(4-hydroxyphenyl) chromane-7-ol-4-*O*-sulfate (Clog*P*, 1.21) based on retention times, fragmentation behaviors, and corresponding Clog*P* values.

Metabolites M44 and M48, detected at 7.64 and 8.33 min, respectively, possessed the same [M−H]^−^ ions at *m*/*z* 271.0600 (C_15_H_11_O_5_, error ≤ ±1.00 ppm). In the ESI-MS^2^ spectra of M44, the occurrence of DPIs at *m*/*z* 165 (generated by RDA rearrangement occurring at positions 2 and 3) and *m*/*z* 151 (yielded by RDA rearrangement occurring at positions 1 and 3) were observed, and thus, M44 was tentatively identified as 5, 7-dihydroxy-3-(4-hydroxyphenyl) chroman-4-one. As for M48, the base peak ion at *m*/*z* 253 resulted from a neutral loss of H_2_O, which indicated the reduction probably occurred on carbonyl attached to position C4. Therefore, M48 was tentatively identified as 3-(4-hydroxyphenyl)-4*H*-chromene-4,5,7-triol.

M42 eluted at 6.73 min gave rise to the [M−H]^−^ ion at *m*/*z* 285.0760 (C_16_H_13_O_5_, 0.30 ppm). It was 14 Da more than that of M44 or M48, suggesting it could be methylation product of M44 or M48. In its ESI-MS^2^ spectrum, the product ion at *m*/*z* 270 yielded by neutral loss of CH_3_ that indicated the occurrence of methylation. The product ions at *m*/*z* 164 and *m*/*z* 150 in its ESI-MS^3^ spectrum were consistent with the fragmentation behavior of M44, and further indicated that methyl group was introduced into the B-ring. However, the actual reaction site could not be determined at the current stage, therefore, M42 was tentatively identified as a methylation product of M44. 

Metabolites M43 and M54 respectively eluted at 6.83 and 9.65 min gave rise to the [M−H]^−^ ions at *m*/*z* 287.0922 (C_16_H_15_O_5_, 0.90 ppm) and *m*/*z* 287.0921 (C_16_H_15_O_5_, 0.79 ppm). They were 30 Da more than that of M53. Moreover, the DPI at *m*/*z* 272 formed by neutral loss of CH_3_ from *m*/*z* 287 in their ESI-MS^2^ spectra indicated the occurrence of a methylation reaction. In the ESI-MS^2^ spectrum of M43, another two DPIs at *m*/*z* 151 and *m*/*z* 137 formed by the RDA rearrangement were observed, which indicated that methyl group was introduced into the B-ring as well as the hydroxylation. In the ESI-MS^2^ spectrum of M54, the DPI at *m*/*z* 124 was yielded owing to RDA rearrangement, which indicated that methylation occurred on the A-ring as well as the hydroxylation, but in both cases the actual reaction sites could not be determined, thus, M43 and M54 were tentatively identified as hydroxylation and methylation products of M53.

#### 2.4.4. Identification of Equol Species Metabolites

Three sequential chromatographic peaks, M26, M33 and M46 were eluted at 5.18, 5.80 and 8.24 min, respectively. They yielded accurate [M−H]^−^ at *m*/*z* 241.0867, *m*/*z* 241.0867, and *m*/*z* 241.0868 (0.87, 0.87 and 0.89 ppm, C_15_H_13_O_3_). The DPI at *m*/*z* 135 (yielded by RDA rearrangement occurring at positions 2 and 3) and *m*/*z* 121 (generated by RDA rearrangement occurring at positions 1 and 3) were observed. Metabolite M51 was eluted at 8.95 min with its [M + H]^+^ ion at *m*/*z* 243.1015 (C_15_H_15_O_3_, −2.80 ppm). In its ESI-MS^2^ spectrum, abundant DPIs at *m*/*z* 123 (RDA rearrangement occurring at positions 1 and 3) and *m*/*z* 107 (RDA rearrangement occurrig at positions 1 and 4) were observed, which were identical with the detected metabolites in negative ion mode and published literature [31]. Therefore, M26, M33, M46, and M51 were tentatively identified as equol or its positional isomers [32,33].

Metabolite M27 gave rise to the [M−H]^−^ ion at *m*/*z* 417.11911 (C_21_H_21_O_9_, 1.10 ppm) with a retention time of 5.18 min. It was 176 Da more than that of equol, and DPIs at *m*/*z* 241 ([M-H-GluA]^−^) and *m*/*z* 175 ([GluA-H]^−^) in its ESI-MS^2^ spectrum fully validated the presence of a glucuronide group. Therefore, M27 was tentatively identified as equol-*O*-glucuronide.

Metabolite M52 eluted at 9.65 min possessed [M + H]^+^ ion at *m*/*z* 241.0854 (C_15_H_13_O_3_, −0.49 ppm). This was 2 Da less than that of equol in positive ion mode, which suggested that it might be dehydrogenation product of equol. Furthermore, DPI at *m*/*z* 147 ([M-H-C_6_H_6_O]^−^) confirmed that the reduction reaction might occur on positions 1 and 3 in the C-ring, and thus, M52 was tentatively identified as 3-(4-hydroxyphenyl)-4*H*-chromen-7-ol.

#### 2.4.5. Identification of Decarbonylation Species Metabolites

Metabolites M18 and M36 possessed the same [M + H]^+^ ions at *m*/*z* 227.0701 (C_14_H_11_O_3_, error ≤ ±1.00 ppm). In their ESI-MS^2^ spectra, the product ion at *m*/*z* 199 was produced through neutral loss of CO moiety from the parent ion of daidzein with no more characteristic product ions being observed. They were tentatively identified as decarbonylation products of daidzein on the basis of accurate mass weight and elemental composition.

### 2.5. Proposed Metabolic Pathways of Daidzein

**Figure 5:**
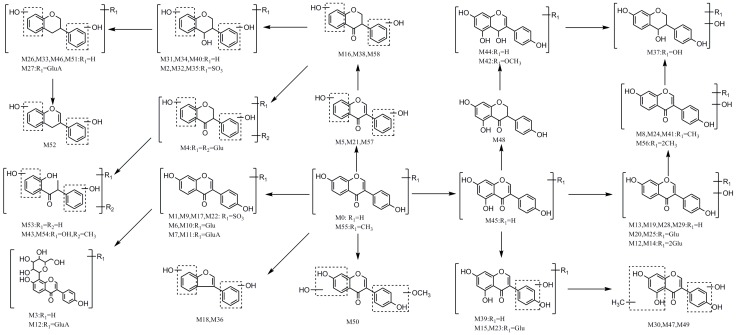
The proposed daidzein metabolic patterns in vivo.

In this paper, a total of 59 daidzein metabolites (prototype compound included) with different structures were observed and identified in rats. The proposed metabolic pathways of daidzein are illustrated in Figure 5. There are cardinal corresponding bio-reactions found in vivo, which can be divided into several categories, including dehydration, hydrogenation, methylation, dimethylation, glucuronidation, glucosylation, sulfonation, ring-cleavage and their composite reactions. In addition, it should be noted that some unusual products were detected. For example, the carbonyl group in C-ring of M31, M32, M34 and M40 was transformed into a hydroxyl during in vivo biotransformation. Then, a dehydroxylation of this newly generated hydroxyl group occurred, and thus metabolites M26, M27, M33, and M46 were attributed to iso-flavonol-like compounds. In addition, metabolites M3 and M12 were attributed to C-glycoside kind compounds in this study, which have not been reported ever before.

## 3. Discussion

The metabolic profile of daidzein in urine and plasma was studied following oral administration of daidzein to SD rats. Using UHPLC-LTQ-Orbitrap MS combined with multiple off-line data-mining methods including MMDFs, HREIC, DPIs, and NLFs analysis, a total of 59 metabolites as well as prototype compounds were identified. Since compounds with larger Clog*P* values will have a longer retention times in a reversed phase (RP) chromatographic system, the structures of daidzein metabolites were tentatively elucidated. In order to eliminate the influence of the diet, all of the detected metabolites were searched using HREICs in the MS spectra of control group samples, and no metabolite was detected in control samples after 12 h ambrosia. Our results indicated that the metabolic pathways should be principally divided into dehydration, hydrogenation, methylation, and dimethylation, and those metabolites could be conjugated subsequently by glucuronidation, glucosylation, and sulfonation. Actually, as a final metabolite of soy isoflavones, equol plays a vital role in the prevention of cardiovascular disease, breast, and prostate cancer. In addition, other metabolites, such as genistein, genistin and daidzin, also show many significant bioactivities. This study also provided a valuable and latest information in the field of the metabolic fate of daidzein, which is indispensable for further understanding the mechanism of its effects and safety monitoring of daidzein.

## 4. Materials and Methods

### 4.1. Chemicals and Reagents

Five reference standards including daidzein, daidzin, puerain, genistein, and genistin were purchased from Chengdu Must Biotechnology Co. Ltd. (Sichuan, China). Their structures were fully ascertained by comparing the spectra data with published literature values. Their purities were all no less than 98% by HPLC-UV analysis. HPLC grade acetonitrile, methanol, and formic acid (FA) were used and supplied by Fisher Scientific (Fair Lawn, NJ, USA). Grace Pure™ SPE C18-Low solid-phase extraction (SPE) cartridges (200 mg/3 mL, 59 μm, 70 Å) were obtained from Grace Davison Discovery Science™ (Deerfield, IL, USA). Deionized water was freshly prepared using a Milli-Q Gradient Å 10 System (Millipore, Billerica, MA, USA). All other chemicals were of analytical grade and commercially available at the work station, Beijing Chemical Works (Beijing, China).

### 4.2. Animal and Drug Administration

Ten male SD rats weighing 200–220 g were provided by Beijing Weitong Lihua Experimental Animals Company (Beijing, China). The animals were housed individually at a constant temperature of 22 ± 1 °C and humidity of 50 ± 10% with free food intake and water consumption for a week for acclimatization. After that, all rats were randomly divided into Drug Group (for test plasma and urine, *n* = 5) and Control Group (for blank plasma and urine, *n* = 5). Before the experiment, all rats were fasted for 12 h with free access to water. The animal facilities and protocols were approved by the institutional Animal Care and Use Committee in Beijing University of Chinese Medicine. All procedures used were carried out according to Guide for the Care and Use of Laboratory Animals of the US National Institutes of Health.

### 4.3. Sample Collection

Daidzein was suspended in 0.5% sodium carboxymethyl cellulose (CMC-Na) solution and orally administered to Drug group at a dose of 200 mg/kg body weight. Equivalent 0.5% CMC-Na solution was administered by oral gavage to the Control group.

#### 4.3.1. Plasma Sample Collection

After oral administration, all rats were put into metabolic cages. The rats in the Drug group and Control group were respectively taken to obtain test and blank blood. All the blood samples (0.5 mL) were taken from the suborbital venous plexus of rats at 0.5, 1, 1.5, 2 and 4 h post-administration after oral administration every time. Each sample was centrifuged at 3500 rpm (4 °C) for 15 min to separate plasma. After that, plasma samples from the same group were merged into a collective one. 

#### 4.3.2. Urine Sample Collection

The rats were maintained in metabolic cages to collect urine samples (0–24 h) for test and control urine samples, which were also centrifuged at 3500 rpm (4 °C) for 10 min to exclude the residue. Finally, as described above, all samples from the same group were merged together.

### 4.4. Biological Sample Preparation

An approach involved protein and solid residue precipitation and concentration was performed to prepare all biological samples. Plasma and urine samples (1 mL) were respectively added into SPE cartridge, which was pretreated with methanol (5 mL) and deionized water (5 mL). And then, SPE cartridge was successively washed with deionized water (5 mL) and methanol (3 mL). The methanol eluate was collected and evaporated in nitrogen at room temperature. The residue was then redissolved in 80 μL of acetonitrile/water (5:95, *v*/*v*) and centrifuged at 14,000 rpm (4 °C) for 30 min. The supernatant was used for instrumental analysis.

### 4.5. Instruments and Conditions

All the LC-MS analyses were performed on an UHPLC-LTQ-Orbitrap mass spectrometer (Thermo Scientific, Bremen, Germany) equipped with an ESI source (Thermo Electron, Bremen, Germany). The chromatographic separation was carried out on a Waters ACQUITY BEH C18 column (2.1 × 100 mm i.d., 1.7 μm; Waters Corporation, Milford, MA, USA) with column temperature set at 30 °C. Acetonitrile (solvent B) and 0.1% FA aqueous solution (solvent A) were used as mobile phases. The flow rate was 0.3 mL/min applied with a linear gradient set as follows: 0–2 min, 5–20% B; 2–27 min, 20–85% B; 27–30 min, 85% B. The injection volume was 2 μL.

The optimized operating parameters in both negative and positive ion modes were as set as follows: capillary voltage of 35 V, electrospray voltage of 3.0 kV, capillary temperature of 350 °C, sheath gas flow rate of 40 (arbitrary units), auxiliary gas flow rate of 20 (arbitrary units), and tube lens of 110 V. Metabolites were detected using full-scan MS analysis from *m*/*z* 100–800 at a resolving power of 70,000. Data-dependent ESI-MS^2^ analyses were triggered by the three most-abundant ions from the precursor ions while ESI-MS^3^ analyses of the most-abundant product ions were followed. Collision-induced dissociation (CID) was performed with an isolation width of 2.0 Da. The collision energy was set to 40%.

In the full scan (FS) experiment, HRMS data were recorded at mass resolving power of 70,000 full width at half maximum (FWHM, calculated for *m*/*z* 200). To minimize the total analysis time, data-dependent MS/MS scanning to trigger fragmentation spectra of target ions was performed. The collision energy for collision induced dissociation (CID) was adjusted to 40% of maximum. The dynamic exclusion (DE) to prevent repetition was employed, and the repeat count was set at 5 with the dynamic repeat time at 30 s and dynamic exclusion duration at 60 s. In addition, the parent ion list (PIL)-DE dependent acquisition mode was also employed as a complementary method to obtain MS*^n^* stage of the obtained datasets [15].

### 4.6. Data Processing

A Thermo Xcalibur 2.1 workstation (Thermo Scientific) was adopted for acquiring and processing HR-ESI-MS^1^ and MS*^n^* data. To obtain as many product ions of daidzein metabolites as possible, the peaks detected with intensity over 10,000 for negative ion mode and 50,000 for positive ion mode were selected for further structural characterization. The chemical formula for all parent ions were calculated from accurate mass using a formula predictor with the parameters set as followings: C [6–35], H [5–50], O [0–15], S [0–5], N [0–5], and ring double bond (RDB) equivalent value [0–15]. Meanwhile, MetWorks (Version 1.3) and Mass Frontier (Version 8.0) software (Thermo Scientific, Waltham, MA, USA) were utilized for mass fragmentation behaviors analysis, structural elucidation, and chromatographic peaks extraction.

## Figures and Tables

**Table 1:** Summary of daidzein metabolites in rat urine and plasma.

Peak	Ion Mode	t_R_/min	Formula	Theoretical Mass *m*/*z*	Experimental Mass *m*/*z*	Error (ppm)	MS/MS Product Ions	Identification/Reactions	U	P
M0	N	6.30	C_15_H_9_O_4_	253.0495	253.0498	0.29	MS^2^[253]: 224(100), 209(99), 225(86), 197(45), 208(32), 196(17), 135(16), 211(15), 223(15), 180(10)	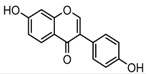	+	+
	P	6.30	C_15_H_11_O_4_	255.0657	255.0644	−0.72	MS^2^[255]: 255(100), 199(27), 137(19), 227(14), 256(13)		+	+
M1	P	3.36	C_15_H_11_O_7_S	335.0219	335.0227	0.83	MS^2^[335]: 255(100), 243(53), 253(28), 305(27), 223(25), 225(25), 271(17), 265(13), 307(11), 227(11)	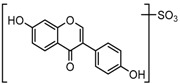	+	−
M2	N	3.36	C_15_H_13_O_7_S	337.0376	337.0385	−1.85	MS^2^[337]: 257(100), 255(15) MS^3^[257]: 215(100), 121(81), 135(74), 239(62), 242(31), 229(25), 173(24), 147(19), 197(18), 149(15), 213(15), 214(10)	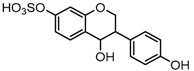	+	−
M3	N	3.46	C_21_H_19_O_9_	415.1023	415.1032	0.88	MS^2^[415]: 295(100) MS^3^[295]: 267(100), 277(3), 293(2)	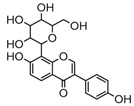	+	−
M4	P	3.72	C_27_H_31_O_14_	579.1708	579.1694	−2.31	MS^2^[579]: 255(100), 481(6)	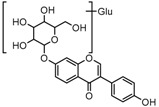	+	−
M5	N	3.85	C_15_H_9_O_4_	253.0495	253.0503	0.84	MS^2^[253]: 253(100), 209(53), 224(46), 225(41), 197(31), 168(18), 193(17), 158(16), 136(16), 149(16), 208(16), 155(15)	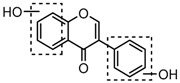	+	−
	P	3.85	C_15_H_11_O_4_	255.0657	255.0646	−0.57	MS^2^[255]: 199(100), 122(94), 137(73), 227(54), 237(24) MS^3^[199]: 181(100), 199(57), 153(23), 157(10), 129(9)		+	−
M6	N	3.85	C_21_H_19_O_9_	415.1023	415.1031	0.76	MS^2^[415]: 253(100), 191(14), 155(12), 252(10), 148(10), 164(10), 314(10)	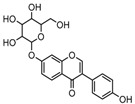	+	−
	P	3.85	C_21_H_21_O_9_	417.1180	417.1170	−0.94	MS^2^[417]: 255(100) MS^3^[255]: 199(100), 227(49), 137(34), 145(23), 237(10), 153(7), 255(7), 211(5), 169(5), 181(4), 155(4)		+	−
M7	N	3.85	C_21_H_17_O_10_	429.0815	429.0824	0.87	MS^2^[429]: 253(100), 175(42),	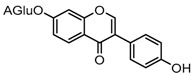	+	−
	P	3.85	C_21_H_19_O_10_	431.0972	431.0963	−0.96	MS^2^[431]: 255(100), 342(13) MS^3^[255]: 199(100), 137(79), 227(57), 237(38), 145(16), 149(9), 165(5), 185(3), 169(3), 141(2), 255(2), 211(2), 129(2), 116(1), 180(1)		+	−
M8	N	3.97	C_16_H_11_O_5_	283.0600	283.0608	0.78	MS^2^[283]: 268(100) MS^3^[268]: 240(100), 239(3), 267(2), 253(2), 238(2)	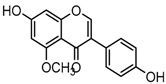	+	−
	P	3.97	C_16_H_13_O_5_	285.0756	285.0752	−0.52	MS^2^[285]: 179(100), 165(18) MS^3^[179]: 81(100), 137(62), 151(46)		+	−
M9	P	4.13	C_15_H_11_O_7_S	335.0219	335.0229	0.98	MS^2^[335]: 227(100), 255(62), 317(57), 273(48), 265(40), 307(23), 253(16), 121(13), 292(13), 149(11), 172(10)	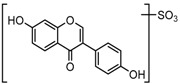	+	−
M10	N	4.26	C_21_H_19_O_9_	415.1023	415.1032	0	MS^2^[415]: 253(100), 252(82), 295(14) MS^3^[253]: 209(100), 225(44), 253(29), 211(26), 197(19), 235(14), 181(12), 185(10), 143(10), 251(8), 135(7)	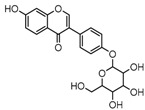	+	−
	P	4.26	C_21_H_21_O_9_	417.1179	417.1171	−1.96	MS^2^[417]: 255(100) MS^3^[255]: 199(100), 237(42), 137(33), 145(15), 171(2)		+	−
M11	N	4.28	C_21_H_17_O_10_	429.0815	429.0827	1.14	MS^2^[429]: 253(100), 175(48), 140(44), 287(39), 147(37), 121(36), 203(36), 235(35), 135(35), 193(31), 119(31), 183(30)	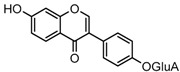	+	−
M12	N	4.36	C_27_H_29_O_15_	593.1500	593.1506	0.89	MS^2^[593]: 269(100), 431(31) MS^3^[269]: 241(100)	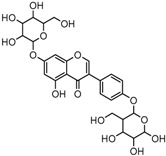	+	−
	P	4.36	C_27_H_31_O_15_	595.1657	595.1643	−2.31	MS^2^[595]: 271(100), 433(57)		+	−
M13	N	4.37	C_15_H_9_O_5_	269.0444	269.0452	0.84	MS^2^[269]: 269(100), 201(56), 225(55), 151(53), 241(38), 224(35), 169(34), 253(32), 252(28), 227(24)	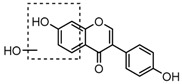	+	−
	P	4.37	C_15_H_11_O_5_	271.0600	271.0594	−0.68	MS^2^[271]: 190(100), 215(95), 153(74), 243(55), 253(53), 149(41), 219(12), 225(12), 271(12)		+	−
M14	P	4.39	C_27_H_31_O_15_	595.1657	595.1644	−2.21	MS^2^[595]: 433(100), 271(75), 433(29), 178(29), 502(26), 253(25), 199(24), 519(23),	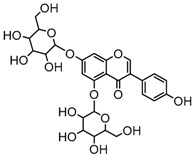	+	−
M15	N	4.50	C_21_H_19_O_11_	447.0921	447.0931	0.98	MS^2^[447]: 285(100) MS^3^[285]: 241(100), 285(97), 199(59), 243(50), 217(38), 257(31), 213(31), 197(31), 267(28), 169(24), 211(24), 201(22), 175(19), 151(9), 239(7), 229(6)	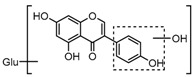	+	+
M16	N	4.56	C_15_H_11_O_4_	255.0652	255.0661	0.94	MS^2^[255]: 135(100), 254(13), 149(10) MS^3^[149]: 121(100)	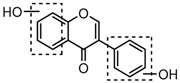	+	−
M17	P	4.56	C_15_H_11_O_7_S	335.0219	335.0232	1.38	MS^2^[335]: 255(100), 227(32), 149(22), 253(15), 271(11) MS^3^[255]: 149(100), 237(13)	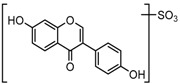	+	−
M18	P	4.58	C_14_H_11_O_3_	227.0701	227.0697	−0.50	MS^2^[227]: 184(100), 209(96), 150(81), 209(52), 199(46), 209(28), 185(28), 199(19), 159(16), 183(15), 209(14), 123(14)	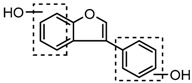	+	−
M19	N	4.61	C_15_H_9_O_5_	269.0444	269.0453	0.93	MS^2^[269]: 269(100), 151(64), 225(61), 201(51), 224(41), 197(34), 253(27), 241(26), 199(12), 239(12), 240(11)	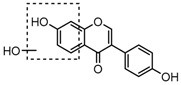	+	−
	P	4.61	C_15_H_11_O_5_	271.0600	271.0595	−0.59	MS^2^[271]: 153(100), 215(73), 243(60), 253(33), 149(26), 145(18), 159(15), 271(12)		+	−
M20	N	4.61	C_21_H_19_O_10_	431.0972	431.0983	1.06	MS^2^[431]: 268(100), 269(78), 311(11) MS^3^[268]: 267(100), 239(83), 240(48), 226(43), 223(42), 195(20), 241(19), 225(18), 212(17), 201(10)	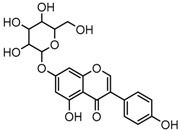	+	−
	P	4.61	C_21_H_21_O_10_	433.1129	433.1118	−2.48	MS^2^[433]: 271(100), 392(11) MS^3^[271]: 153(100), 215(72), 243(55), 145(22), 253(21), 149(21), 271(20), 225(19), 159(14), 199(12), 187(11)		+	−
M21	N	4.87	C_15_H_9_O_4_	253.0495	253.0504	0.92	MS^2^[253]: 211(100), 209(81), 224(68), 225(62), 135(35), 197(30), 117(16)	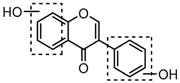	+	−
M22	N	4.87	C_15_H_9_O_7_S	333.0063	333.0071	2.46	MS^2^[333]: 253(100) MS^3^[253]: 211(100), 209(78), 225(57), 224(36), 135(27), 253(26), 251(20), 197(19), 117(18), 223(12), 133(11)	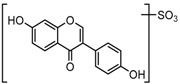	+	−
	P	4.87	C_15_H_11_O_7_S	335.0219	335.0214	−0.55	MS^2^[335]: 255(100) MS^3^[225]: 137(100), 227(77), 199(72), 237(46), 198(26), 255(19), 181(17), 153(14), 171(8), 119(7), 145(6), 209(5)		+	−
M23	N	4.87	C_21_H_19_O_11_	447.0921	447.0931	0.95	MS^2^[447]: 284(100), 285(82), 327(25), 255(21), 270(12), 200(12), 269(11), 124(11)	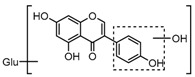	+	−
M24	N	4.94	C_16_H_11_O_5_	283.0600	283.0608	0.78	MS^2^[283]: 268(100), 151(12) MS^3^[268]: 240(100), 224(6), 267(5), 239(4), 163(2)	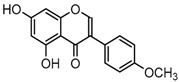	+	−
	P	4.94	C_16_H_13_O_5_	285.0756	285.0751	−0.59	MS^2^[285]: 165(100), 191(17), 207(11) MS^3^[165]: 137(100), 123(79), 109(21)		+	−
M25	N	5.01	C_21_H_19_O_10_	431.0972	431.0983	1.03	MS^2^[431]: 268(100), 269(31) MS^3^[268]: 267(100), 240(99), 239(45), 224(38), 199(22), 223(19), 211(7), 251(7)	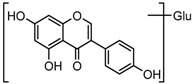	+	−
M26	N	5.18	C_15_H_13_O_3_	241.0858	241.0867	0.87	MS^2^[241]: 212(100), 135(42), 121(19), 119(16)	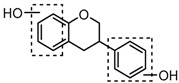	+	−
	P	5.18	C_15_H_15_O_3_	243.1015	243.1010	−0.51	MS^2^[243]: 123(100), 107(44), 137(9) MS^3^[123]: 95(100)		+	−
M27	N	5.18	C_21_H_21_O_9_	417.1180	417.1191	1.10	MS^2^[417]: 175(100), 241(24), 113(23), 399(20), 181(13) MS^3^[175]: 113(100)	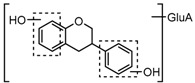	+	+
M28	N	5.27	C_15_H_9_O_5_	269.0444	269.0454	0.96	MS^2^[269]: 241(100), 213(28), 253(11), 251(10) MS^3^[241]: 213(100), 197(18)	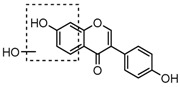	+	−
	P	5.27	C_15_H_11_O_5_	271.0600	271.0594	−0.62	MS^2^[271]: 215(100), 225(99), 153(98), 253(75), 243(38), 181(19), 161(16), 197(16), 253(11), 271(10) MS^3^[215]: 197(100), 169(24), 153(20), 187(15), 159(8), 215(6), 141(5), 173(3)		+	−
M29	N	5.51	C_15_H_9_O_5_	269.0444	269.0453	0.90	MS^2^[269]: 225(100), 213(62), 241(61), 240(47), 151(31), 224(27), 195(21), 181(20), 173(19), 253(14), 209(13)	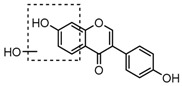	+	−
	P	5.51	C_15_H_11_O_5_	271.0600	271.0594	−0.65	MS^2^[271]: 215(100), 243(84), 253(78), 153(63), 225(43), 253(37), 145(35), 121(30), 197(27), 15(19), 149(19), 151(18)		+	−
M30	N	5.60	C_16_H_11_O_6_	299.0550	299.0558	0.84	MS^2^[299]: 284(100) MS^3^[284]: 256(100), 240(30), 269(7), 267(7), 228(6), 150(4)	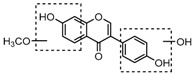	+	−
	P	5.60	C_16_H_13_O_6_	301.0701	301.0698	−0.78	MS^2^[301]: 286(100), 167(51), 245(49), 161(38), 255(33), 273(30), 283(29), 241(19), 283(11) MS^3^[286]: 258(100), 229(30), 153(15), 212(15), 213(8), 240(7), 200(6), 285(6), 269(6), 188(5), 184(5)		+	−
M31	N	5.70	C_15_H_13_O_4_	257.0808	257.0817	0.90	MS^2^[257]: 137(100), 161(44), 228(41), 147(22), 242(21), 214(21), 129(15), 189(14), 101(13), 229(13)	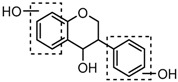	+	−
M32	N	5.79	C_15_H_13_O_7_S	337.0376	337.0383	0.71	MS^2^[337]: 257(100), 217(20) MS^3^[257]: 135(100), 137(92), 121(27), 147(16), 239(13), 109(10), 187(9), 103(6), 215(5), 151(2)	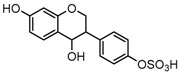	+	−
M33	N	5.80	C_15_H_13_O_3_	241.0858	241.0867	0.81	MS^2^[241]: 121(100), 135(53), 119(18)	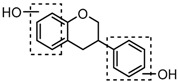	+	−
M34	N	5.90	C_15_H_13_O_4_	257.0808	257.0815	0.74	MS^2^[257]: 137(100), 121(38), 147(17), 109(10)	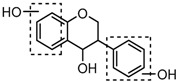	+	−
M35	N	5.90	C_15_H_13_O_7_S	337.0376	337.0383	0.65	MS^2^[337]: 257(100) MS^3^[257]: 135(100), 121(40), 147(18), 109(9), 137(7), 242(2), 129(1), 197(1), 93(1)101(1)	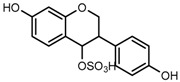	+	−
M36	N	6.30	C_14_H_9_O_3_	225.0546	225.0548	0.20		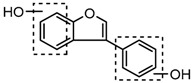	+	−
	P	6.30	C_14_H_11_O_3_	227.0701	227.0698	−0.45	MS^2^[227]: 199(100), 182(95), 157(42), 184(23), 209(22), 181(21), 191(12), 171(11), 209(11), 149(10), 185(9) MS^3^[199]: 154(100), 171(25), 107(13), 143(11), 153(11)		+	−
M37	P	6.39	C_15_H_15_O_5_	275.0908	275.0908	−0.55	MS^2^[275]: 151(100) MS^3^[151]: 123(100), 151(23), 141(8)	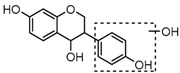	+	−
M38	N	6.40	C_15_H_11_O_4_	255.0651	255.0655	0.37	MS^2^[255]: 149(100), 135(18), 254(13) MS^3^[149]: 121(100), 93(11)	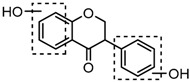	+	−
	P	6.40	C_15_H_13_O_4_	257.08083	257.0802	−0.58	MS^2^[257]: 163(100), 123(58), 256(39), 200(19), 137(14) MS^3^[163]: 135(100), 167(15)		+	−
M39	N	6.52	C_15_H_9_O_6_	285.0393	285.0403	0.98	MS^2^[285]: 257(100), 229(24), 66(11), 217(10), 151(10) MS^3^[257]: 229(100), 146(4), 215(3), 185(2), 137(2)	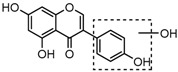	+	−
	P	6.52	C_15_H_11_O_6_	287.0544	287.0546	−0.42	MS^2^[287]: 153(100), 269(59), 241(55), 231(46), 66(23), 259(20), 269(19), 149(12), 213(12), 269(12), 161(10)		+	−
M40	N	6.62	C_15_H_13_O_4_	257.0808	257.0819	1.08	MS^2^[257]: 137(100), 121(44), 147(22), 109(12)	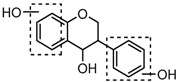	+	−
M41	N	6.62	C_16_H_11_O_5_	283.0600	283.0611	1.00	MS^2^[283]: 268(100) MS^3^[268]: 240(100), 239(9), 224(4), 267(3), 196(2), 184(2)	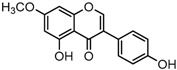	+	−
	P	6.62	C_16_H_13_O_5_	285.0756	285.0749	−0.83	MS^2^[285]: 285(100), 270(72), 286(22), 229(14), 225(13) MS^3^[285]: 270(100), 229(28), 285(25), 225(23), 145(17), 167(17), 257(11), 197(10), 253(7), 267(4), 123(3)		+	−
M42	N	6.73	C_16_H_13_O_5_	285.0757	285.0760	0.30	MS^2^[285]: 270(100), 149(24) MS^3^[270]: 242(100), 164(30), 241(26), 213(8), 151(5)	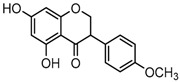	+	−
M43	N	6.83	C_16_H_15_O_5_	287.0913	287.0922	0.90	MS^2^[287]: 151(100), 137(85), 272(82), 135(71), 165(23)	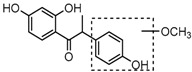	+	−
M44	N	7.64	C_15_H_11_O_5_	271.0600	271.0610	0.94	MS^2^[271]: 165(100), 151(6) MS^3^[165]: 137(100), 121(23), 109(21), 93(6)	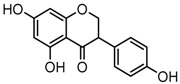	+	−
	P	7.64	C_15_H_13_O_5_	273.0757	273.0751	−0.59	MS^2^[273]: 179(100), 123(58), 153(47), 245(14), 159(11)		+	−
M45	N	8.05	C_15_H_9_O_5_	269.0444	269.0453	0.86	MS^2^[269]: 269(100), 225(49), 181(28), 201(24), 241(20), 151(16)	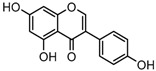	+	−
	P	8.05	C_15_H_11_O_5_	271.0600	271.0591	−0.93	MS^2^[271]: 153(100), 215(79), 243(60), 253(53), 149(45), 153(28), 225(23), 159(19), 197(17), 145(16), 209(16), 165(14)		+	−
M46	N	8.24	C_15_H_13_O_3_	241.0858	241.0868	0.89	MS^2^[241]: 121(100), 135(54), 119(15)	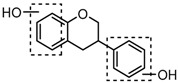	+	−
	P	8.24	C_15_H_15_O_3_	243.1015	243.1008	−0.68	MS^2^[243]: 123(100), 107(39), 133(12), MS^3^[123]: 95(100), 67(18), 79(7)		+	−
M47	N	8.24	C_16_H_11_O_6_	299.0550	299.0555	0.53	MS^2^[299]: 284(100), 239(22), 284(10) MS^3^[284]: 240(100), 256(47), 255(32), 227(29), 239(25), 214(24), 211(15), 267(13)	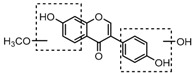	+	−
	P	8.24	C_16_H_13_O_6_	301.0701	301.0697	−0.88	MS^2^[301]: 286(100)		+	−
M48	N	8.33	C_15_H_11_O_5_	271.0601	271.0608	0.78	MS^2^[271]: 253(100), 270(41), 225(17), 151(15), 215(15), 227(12), 125(10)	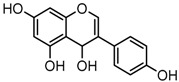	+	−
M49	N	8.44	C_16_H_11_O_6_	299.0550	299.0557	0.75	MS^2^[299]: 284(100) MS^3^[284]: 256(100), 227(27), 255(24), 284(18), 212(13), 228(12), 239(11), 211(11),	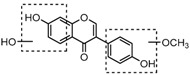	+	−
	P	8.44	C_16_H_13_O_6_	301.0701	301.0699	−0.72	MS^2^[301]: 286(100), 269(56), 241(18) MS^3^[286]: 258(100), 153(73), 229(9), 269(9), 285(7), 230(5), 134(3), 259(3), 202(2), 268(2), 212(1)		+	−
M50	N	8.74	C_16_H_11_O_6_	299.0550	299.0558	0.84	MS^2^[299]: 284(100), 254(60), 190(11), 255(11), MS^3^[284]: 256(100), 227(91), 239(84), 255(57), 240(54), 283(39), 188(22), 211(21), 283(20), 211(21), 283(20)	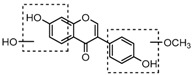	+	−
	P	8.74	C_16_H_13_O_6_	301.0701	301.0699	−0.72			+	−
M51	P	8.95	C_15_H_15_O_3_	243.1015	243.1015	−2.80	MS^2^[243]: 123(100), 133(63), 107(15), 225(11) MS^3^[123]: 95(100)	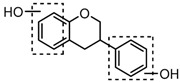	+	−
M52	N	9.65	C_15_H_11_O_3_	239.0702	239.0710	0.79	MS^2^[239]: 147(100), 195(28), 210(24), 197(17), 211(14), 132(12), 121(12), 105(12)	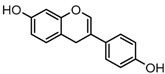	+	−
	P	9.65	C_15_H_13_O_3_	241.0854	241.0854	−0.49	MS^2^[241]: 213(100) MS^3^[213]: 119(100), 195(73), 167(62), 211(47), 171(45), 157(31), 185(25), 169(13)		+	−
M53	N	9.65	C_15_H_13_O_4_	257.0808	257.0817	0.90	MS^2^[257]: 239(100), 109(67), 163(40), 242(29), 213(29), 148(21), 147(18), 136(15), 224(14) MS^3^[239]: 145(100), 223(54), 221(20), 197(14), 211(13), 171(11), 195(10), 133(10), 169(10), 119(7)	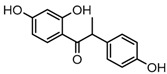	+	−
	P	9.65	C_15_H_15_O_4_	259.0959	259.0960	−0.48	MS^2^[259]: 149(100), 165(35), 121(11) MS^3^[149]: 121(100)		+	−
M54	N	9.65	C_16_H_15_O_5_	287.0913	287.0921	0.79	MS^2^[287]: 272(100) MS^3^[272]: 124(100)	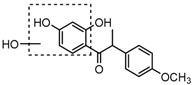	+	−
	P	9.65	C_16_H_17_O_5_	289.1071	289.1064	−0.57	MS^2^[289]: 149(100), 271(77), 121(37), 195(16), 229(12) MS^3^[149]: 121(100), 120(39)		+	−
M55	N	9.93	C_16_H_11_O_4_	267.0652	267.0660	0.89	MS^2^[267]: 252(100) MS^3^[252]: 208(100), 223(97), 224(97), 251(96), 134(29)	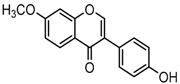	+	−
	P	9.93	C_16_H_13_O_4_	269.0808	269.0802	−0.64	MS^2^[269]: 254(100), 237(44), 213(33), 253(13) MS^3^[254]: 237(100), 226(15), 253(14), 136(5), 254(3)		+	−
M56	P	10.30	C_17_H_15_O_5_	299.0920	299.0904	−0.92	MS^2^[299]: 284(100), 166(22), 243(19), 239(12), 271(11) MS^3^[284]: 256(100), 166(30), 255(22), 267(14), 269(8)	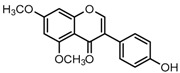	+	−
M57	N	11.76	C_15_H_9_O_4_	253.0495	253.0505	1.02	MS^2^[253]: 209(100), 225(42), 168(38), 180(38), 145(38), 137(37), 141(36), 235(34), 130(32), 103(32), 121(31), 119(31)	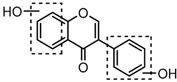	+	−
	P	11.76	C_15_H_11_O_4_	255.0657	255.0646	−0.55	MS^2^[255]: 199(100)137(67), 227(59), 237(24), 145(12) MS^3^[199]: 181(100), 171(30), 153(16), 169(9), 199(6)		+	−
M58	N	12.11	C_15_H_11_O_4_	255.0652	255.0661	1.01	MS^2^[255]: 149(100), 135(35), 254(30) MS^3^[149]: 121(100)	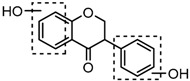	+	−

Note: t_R_: retention time; U: urine; P: plasma; +: detected; −: undetected.

## Supplementary Materials

The supplementary materials are available online.
