# Supplementary material for: Rapid Screening and Identification of Daidzein Metabolites in Rats Based on UHPLC-LTQ-Orbitrap Mass Spectrometry Coupled with Data-Mining Technologies
Source: Molecules. 2018 Jan 12;23(1):151. doi: 10.3390/molecules23010151 (PMC6017279; doi:10.3390/molecules23010151)
Supplement: Supplementary file 1 [file molecules-23-00151-s001.pdf]

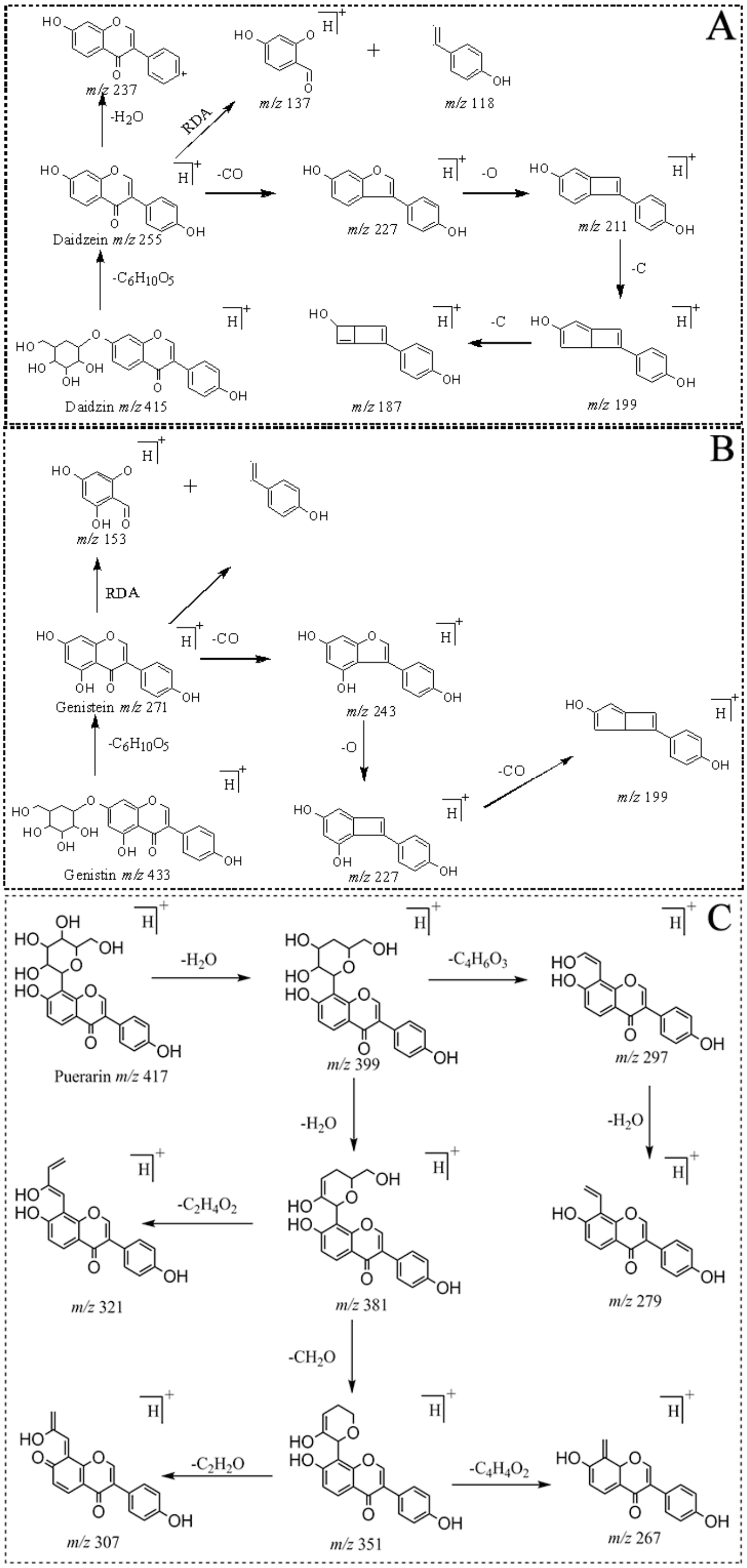

Figure S1. The mass fragmentation behavior of daidzein, daidzein, genistein, genistin, and puerarin in positive ion mode.

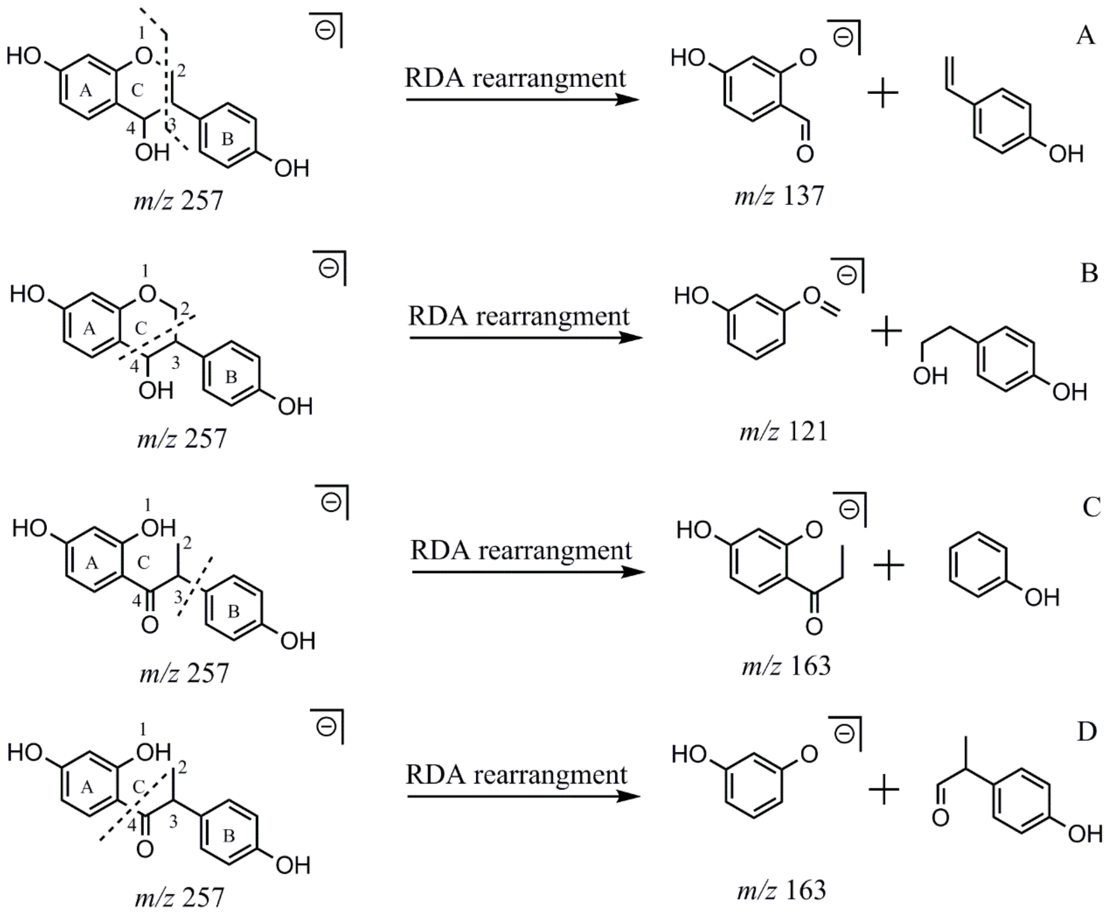

Figure S2. RDA rearrangements in MS/MS spectra of daidzein metabolites.
